# Exercise training and NR supplementation to improve muscle mass and fitness in adolescent and young adult hematopoietic cell transplant survivors: a randomized controlled trial {1}

**DOI:** 10.1186/s12885-022-09845-1

**Published:** 2022-07-19

**Authors:** Minkeun Song, Saro H. Armenian, Rusha Bhandari, Kyuwan Lee, Kirsten Ness, Mary Putt, Lanie Lindenfeld, Saro Manoukian, Kristin Wade, Anna Dedio, Tati Guzman, Isabella Hampton, Kimberly Lin, Joseph Baur, Shana McCormack, Sogol Mostoufi-Moab

**Affiliations:** 1 Division of Oncology, Children’s Hospital of Philadelphia, 3401 Civic Center Boulevard, Philadelphia, PA 19104-4319 USA; 2 Department of Population Sciences, City of Hope, 1500, East Duarte Road, Duarte, CA 91010-3000 USA; 3 Department of Pediatrics, City of Hope, 1500, East Duarte Road, Duarte, CA 91010-3000 USA; 4 Epidemiology and Cancer Control, St. Jude Children’s Research Hospital, 262 Danny Thomas Place, Memphis, TN 38105-3678 USA; 5 Department of Biostatistics, Epidemiology & Informatics, University of Pennsylvania Perelman School of Medicine, 3400 Civic Center Blvd, Philadelphia, PA 19104-5156 USA; 6 Department of Diagnostic Radiology, City of Hope, 1500, East Duarte Road, Duarte, CA 91010-3000 USA; 7 Division of Endocrinology, Children’s Hospital of Philadelphia, 3401 Civic Center Boulevard, Philadelphia, PA 19104-4319 USA; 8 Cardiac Center, Children’s Hospital of Philadelphia, 3401 Civic Center Boulevard, Philadelphia, PA 19104-4319 USA; 9 Department of Physiology, University of Pennsylvania Perelman School of Medicine, 3400 Civic Center Blvd, Philadelphia, PA 19104-5156 USA

**Keywords:** Hematopoietic cell transplantation, Survivors, Sarcopenia, Exercise intervention, NAD + precursor

## Abstract

**Background:**

Advances in hematopoietic cell transplantation (HCT) have led to marked improvements in survival. However, adolescents and young adults (AYAs) who undergo HCT are at high risk of developing sarcopenia (loss of skeletal muscle mass) due to the impact of HCT-related exposures on the developing musculoskeletal system. HCT survivors who have sarcopenia also have excess lifetime risk of non-relapse mortality. Therefore, interventions that increase skeletal muscle mass, metabolism, strength, and function are needed to improve health in AYA HCT survivors. Skeletal muscle is highly reliant on mitochondrial energy production, as reflected by oxidative phosphorylation (OXPHOS) capacity. Exercise is one approach to target skeletal muscle mitochondrial OXPHOS, and in turn improve muscle function and strength. Another approach is to use “exercise enhancers”, such as nicotinamide riboside (NR), a safe and well-tolerated precursor of nicotinamide adenine dinucleotide (NAD^+^), a cofactor that in turn impacts muscle energy production. Interventions combining exercise with exercise enhancers like NR hold promise, but have not yet been rigorously tested in AYA HCT survivors.

**Methods/design:**

We will perform a randomized controlled trial testing 16 weeks of in-home aerobic and resistance exercise and NR in AYA HCT survivors, with a primary outcome of muscle strength via dynamometry and a key secondary outcome of cardiovascular fitness via cardiopulmonary exercise testing. We will also test the effects of these interventions on i) muscle mass via dual energy x-ray absorptiometry; ii) muscle mitochondrial OXPHOS via an innovative non-invasive MRI-based technique, and iii) circulating correlates of NAD^+^ metabolism via metabolomics. Eighty AYAs (ages 15-30y) will be recruited 6–24 months post-HCT and randomized to 1 of 4 arms: exercise + NR, exercise alone, NR alone, or control. Outcomes will be collected at baseline and after the 16-week intervention.

**Discussion:**

We expect that exercise with NR will produce larger changes than exercise alone in key outcomes, and that changes will be mediated by increases in muscle OXPHOS. We will apply the insights gained from this trial to develop individualized, evidence-supported precision initiatives that will reduce chronic disease burden in high-risk cancer survivors.

**Trial registration:**

ClinicalTrials.gov, NCT05194397. Registered January 18, 2022, https://clinicaltrials.gov/ct2/show/NCT05194397 {2a}.

**Supplementary Information:**

The online version contains supplementary material available at 10.1186/s12885-022-09845-1.

## Background

Advances in hematopoietic cell transplantation (HCT) have led to 10% improvements in survival each decade since the 1980s [1]. Research in the growing population of long-term HCT survivors has highlighted the chronic and debilitating co-morbidities associated with toxicity from pre-HCT exposures, HCT conditioning, immunosuppression, and graft versus host disease (GvHD) [2]. Adolescents and young adults (AYAs) who undergo HCT are at an especially high risk of developing sarcopenia (loss of skeletal muscle mass), a consequence of HCT-related exposures on the developing musculoskeletal system [3]. HCT-associated risk factors for sarcopenia include inflammation related to GvHD, treatment-related myopathy (e.g., from glucocorticoids), undernutrition, physical inactivity, and/or de novo co-morbidities (e.g., hypogonadism, growth hormone deficiency) that develop during or shortly after HCT [4]. In AYAs undergoing HCT, we have shown that sarcopenia occurs earlier than would be expected from advancing age alone, likely because the AYA age range (15-30y) represents a critical window for attainment of peak muscle mass. We have also shown that HCT survivors who have sarcopenia have twice the risk of non-relapse mortality as compared to survivors without sarcopenia, which may be mediated in part by an excess incidence of premature cardiovascular disease (CVD) [5]. Therefore, evidence-based interventions are needed that will increase skeletal muscle mass, metabolism, strength, and function and ultimately improve health outcomes in AYA HCT survivors (Table 1).

**Table 1:** World Health Organization Trial Registration Data Set {2b}

**Data category**	**Information** {2b}
Primary registry and trial identifying number	ClinicalTrials.gov NCT05194397
Date of registration in primary registry	January 18, 2022
Secondary identifying numbers	20–017,320 1R01CA254955-01A1 (U.S. NIH Grant/Contract)
Source(s) of monetary or material support	National Cancer Institute (NCI)
Primary sponsor {5b}	National Cancer Institute Sponsor’s Reference: 1R01CA254955-01A1 Contact name: Frank Perna, Ph.D Address: 37 Convent Dr, Bethesda, MD 20,814 Telephone: 240–276-6782 Email: pernafm@mail.nih.gov
Secondary sponsor(s)	
Contact for public queries	Sogol Mostoufi-Moab, MD, MSCE 267–426-6725 moab@chop.edu
Contact for scientific queries	Sogol Mostoufi-Moab, MD, MSCE 267–426-6725 moab@chop.edu
Public title	Exercise Training and NR Supplementation Trial to Improve Fitness in AYA HCT Survivors
Scientific title	Intensive Tailored Exercise Training With NAD + Precursor Supplementation to Improve Muscle Mass and Fitness in Childhood Cancer Survivors
Countries of recruitment	United States of America
Health condition(s) or problem(s) studied	Acute Lymphoblastic Leukemia in Remission, Cancer Survivors
Intervention(s)	Dietary Supplement: Nicotinamide Riboside—GMP-grade 300 mg capsules of the dietary supplement nicotinamide riboside (ChromaDex, Irvine CA) Placebo—same excipients without the active supplement and is generally recognized as safe Other: Exercise Intervention—at-home training sessions and will include aerobic and strengthening components designed to progress persons gradually to 150–300 min of the equivalent of moderate aerobic activity, and twice weekly strengthening over 16 weeks
Key inclusion and exclusion criteria	Ages eligible for study: 15 – 30 years at enrollment, inclusive Sexes eligible for study: both Accepts healthy volunteers: no Inclusion Criteria: Males and females, ages 15–30 years at enrollment; able to understand and speak English; diagnosis of acute leukemia (myeloid, lymphoid) requiring allogeneic HCT; 6–24 months from allogeneic HCT; if female, negative urine/serum pregnancy test and must use an acceptable method of contraception, including abstinence, a barrier method (diaphragm or condom), Depo-Provera, or an oral contraceptive, for the duration of the study; Parental/guardian permission (informed consent) and if appropriate, child assent Exclusion Criteria: Known sensitivity to NR; Concurrent use of any medications, including statins, likely to increase risk of NR toxicity; Active malignancy, investigational agent(s) within 4 wks, or systemic glucocorticoids within 12 wks; Currently meeting public health exercise guidelines; Use of NAD + precursors (supra-physiologic) within 4 weeks; Hemoglobin < 10 g/dL; Platelets < 50 K; Diabetes Mellitus requiring insulin or insulin secretagogue; HbA1C ≥ 8%; Kidney disease (eGFR < 60 ml/min/1.73 m2); Liver disease Alanine aminotransferase/Aspartate aminotransferase(ALT/AST) > 3 × ULN; Limitations in physical function preventing exercise testing/training; Contraindications to MRI; Unstable angina or history of acute myocardial infarction (< 5 days of planned study procedures); Recurrent syncope; Symptomatic severe aortic stenosis; Uncontrolled arrhythmia causing symptoms; Pulmonary embolus < 3 months of study procedures; Thrombosis of lower extremities; Symptomatic moderate or severe persistent asthma based on forced expiratory volume (FEV) from pre-HCT pulmonary function testing; Room air desaturation at rest ≤ 85%; Females: Pregnant or planning pregnancy; Non-cardiopulmonary disorders that may affect exercise performance or be aggravated by exercise (e.g. infection, renal failure, thyrotoxicosis, > moderate graft versus host disease (GVHD) resulting in physical or functional impairment); Parents/guardians or subjects who, in the opinion of the Investigator, may be non-compliant with study schedules or procedures
Study type	allocation: Randomized Intervention Model: Factorial Assignment Intervention Model Description: Randomized, placebo-controlled trial with a 2 × 2 factorial design testing the effects of an NAD + precursor (NR) and exercise Masking: Quadruple (Participant, Care Provider, Investigator, Outcomes Assessor) Masking Description: The participants and the investigator team will be blinded as to the group assignment: NR vs Placebo. All collected data (e.g., questionnaires) will be coded, so initial analysis will be conducted without knowledge of the participant's group status While the assignment of participants to the exercise groups will not be blinded to the participant or majority of the study team, a designated blinded technician will perform the follow-up Cardio Pulmonary Exercise Testing. Follow-Up Cardio Pulmonary Exercise Testing will be performed by a dedicated blinded study team member, an exercise technician, who will not know if the participant was assigned to an arm including the exercise intervention Phase II
Date of first enrolment	June 2022
Target sample size	80
Recruitment status	Recruiting
Primary outcome(s)	Within participant changes in muscle strength (Isometric knee extension, Z-score) [ Time Frame: Baseline to 16 Weeks]
Key secondary outcomes	Within participant change in muscle strength (Ankle Plantarflexion) using Biodex dynamometer [ Time Frame: Baseline to 16 Weeks] Within participant change in grip strength (Hand Grip Dynamometry) [ Time Frame: Baseline to 16 Weeks] Within participant changes in muscle mass (lower leg lean muscle mass by DXA) [ Time Frame: Baseline to 16 Weeks] Within participant changes in post-exercise oxidative phosphorylation capacity (OXPHOS) using non-invasive MRI scanning using creatine chemical exchange saturation transfer (CrCEST) [ Time Frame: Baseline to 16 Weeks] Within participant change in aerobic capacity (VO2 max, Maximal Oxygen Uptake on Cardiopulmonary Exercise Testing). [ Time Frame: Baseline to 16 Weeks]

### Strategies to improve skeletal muscle health

Skeletal muscle is highly reliant on mitochondrial energy production, as reflected by oxidative phosphorylation (OXPHOS) capacity. Thus, one strategy to improve skeletal muscle function is to increase muscle OXPHOS. Exercise (aerobic and resistance training) is a well-established approach to target skeletal muscle mitochondrial OXPHOS, as well as mass, strength, and function, though responsiveness to exercise training interventions may vary in both individuals who are healthy and those who have chronic disease. Moreover, individuals who survive HCT often do not meet minimum public health recommendations for exercise, and also demonstrate significant declines in daily physical activity from pre- to post-HCT [6]. In addition, individuals who develop GvHD are even more likely to be inactive because the disproportionate atrophy that develops in lower extremity and back extensor muscles makes activity more challenging to pursue. The combination of inactivity plus decreased muscle mass further compromises muscle capillary transport and utilization of oxygen, leading to additional impairments in cardiovascular reserve [7]. Thus, targeting physical inactivity along with treatment-related muscle metabolic deficits may produce benefits that are more than additive in sedentary HCT survivors.

Another approach to improve skeletal muscle health is to use an “exercise enhancer” such as precursors of nicotinamide adenine dinucleotide (NAD^+^), a cofactor with myriad roles in cellular metabolism, to recapitulate and/or add to the physiologic benefits of exercise [8, 9]. Nicotinamide riboside (NR) is an NAD^+^ precursor that is consumed as part of the diet and can be administered orally in humans [8]. In preclinical models, NR has been shown to increase muscle OXPHOS capacity and may improve exercise performance [10]. NAD^+^ precursor supplementation with NR has also been shown to increase oxidative metabolism in both muscle [11, 12] and liver [13], and its therapeutic potential is being explored in the wide range of conditions where disordered mitochondrial metabolism occurs, including aging and CVD [14].

Although NR is a niacin analog, unlike niacin, NR is unlikely to produce flushing because it does not activate the niacin receptor (GPR109A) [15]. The pharmacokinetics and pharmacodynamics of NR have been measured in humans [16–18], and safety and toxicity studies in both animals and humans indicate a favorable safety profile that is similar to or better than niacin [18]. Two recent studies in humans found that NR, which is metabolized to nicotinamide, was well tolerated without serious adverse events [19, 20]. Pediatric participants (*n* = 163) who received 1.2 g/m^2^/day of nicotinamide for 5 years had a similar number of serious adverse events as participants receiving placebo (*n* = 166) [21]. A safety study of higher dose (up to 8 g daily) of nicotinamide for up to 8 weeks in individuals with complex neuromuscular disease found it to be generally well-tolerated, with nausea being the predominant side effect observed at the highest doses that resolved spontaneously after dose reduction and/or with anti-nausea treatment [22]. In 12 older men receiving 1 g daily of NR for 21 days, NR was well tolerated, and produced an increase in skeletal muscle NAD^+^ and a decrease in circulating inflammatory cytokines [23]. A longer 12-week trial tested 1,000 mg po twice daily (that is, a total daily dose of 2,000 mg NR) in *N* = 40 healthy sedentary men, and found NR to be well-tolerated over this duration with sustained increases in urinary NAD^+^ metabolites [19] {6b}.

### Rationale for combining exercise with NR to improve muscle health in AYA HCT survivors

**Fig. 1:**
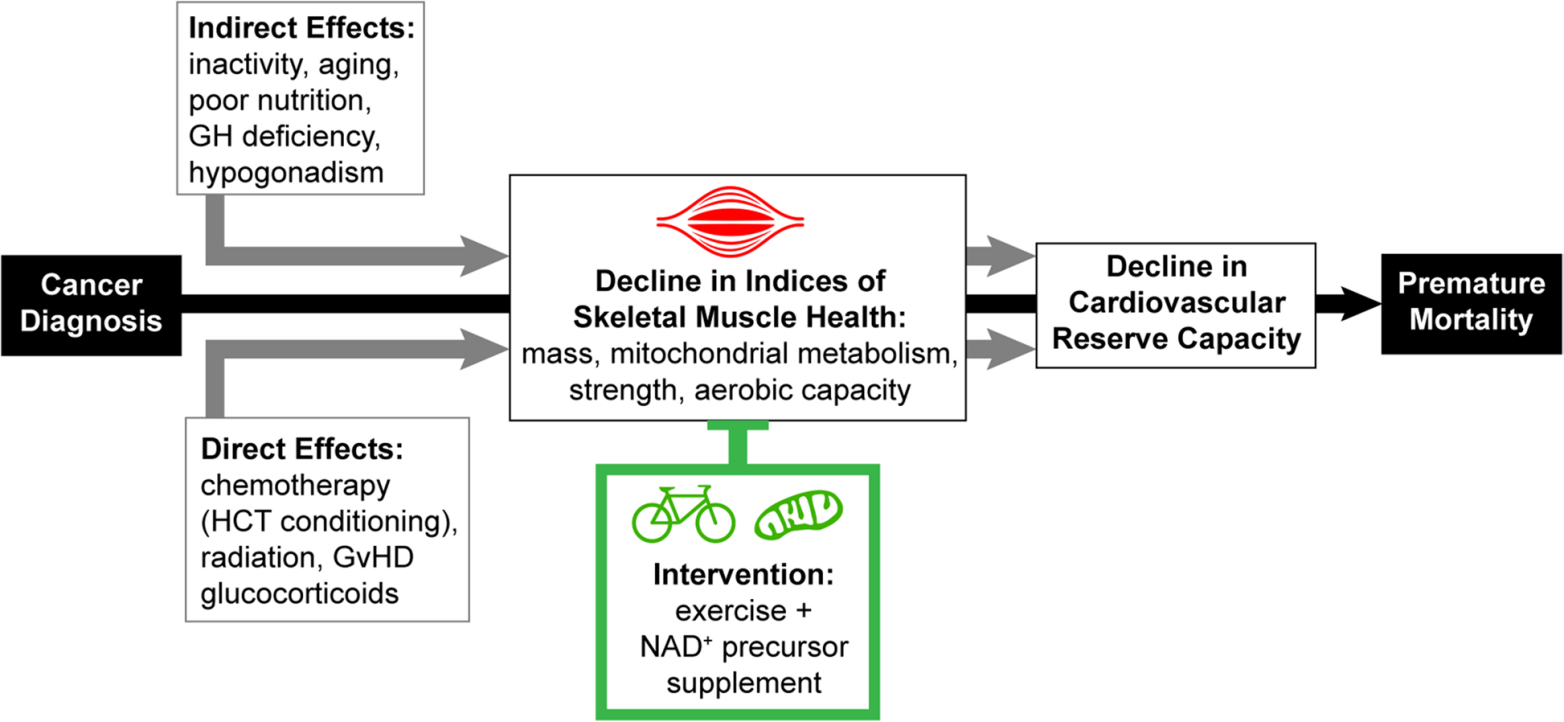
An improvement of overall skeletal muscle health via combination intervention of exercise and NAD + precursor supplementation. Cancer has both direct and indirect effects that compromise cardiovascular reserve capacity, and thus contribute to premature mortality. Both exercise and NAD + precursor therapy (nicotinamide riboside, NR) may increase skeletal muscle mass, mitochondrial OXPHOS capacity, strength, and aerobic capacity. This, in turn, will lead to a lower rate of premature mortality caused by a significant decline in cardiovascular reserve capacity. The figure was generated using Adobe Illustrator (version 22.0)

Exercise and NAD^+^ precursor supplementation via NR are each independently expected to improve muscle mass and strength in HCT survivors. When combined, exercise and NR may even produce benefits that are synergistic (Fig. 1). Exercise requires energy, and NAD^+^ availability promotes mitochondrial ATP production. Indeed, one of the known physiologic adaptations to exercise training in humans is up-regulation of NAD^+^ salvage via increased expression of NAMPT [24]. Studies in preclinical models demonstrate the relevance of this response. For example, mice who have increased NAD^+^ availability gain more exercise capacity in response to exercise training than their counterparts with typical NAD^+^ availability [25]. Also, increased NAD^+^ availability leads to more sirtuin-dependent PGC-1α related mitochondrial biogenesis with training [25]. Recently released physical activity guidelines emphasize the universal benefits of exercise, including for individuals with health problems [26]. However, exercise can require a disproportionate investment of time and effort for those with chronic conditions. Strategies such as NAD^+^ precursor supplementation that enhance the benefits of training would increase the yield of this important investment and could be a valuable addition to our therapeutic arsenal. Therefore, exercise combined with NAD^+^ precursor supplementation may yield additional benefits compared to either alone, but this approach has not been tested in AYA HCT survivors {6a}.

## Methods/design

In this randomized controlled trial, we will prospectively enroll 80 participants status-post HCT at two sites, the Children’s Hospital of Philadelphia (CHOP) (Philadelphia, PA, USA) and the City of Hope Medical Center (COH) (Duarte, CA, USA). There will be 4 arms: exercise intervention with NR supplementation, exercise intervention with placebo, no exercise intervention with NR supplementation, and no exercise intervention with placebo. The individualized exercise intervention will occur in the home, overseen remotely via Telehealth by exercise intervention physiologists from St. Jude Children’s Research Hospital (Memphis, TN, USA) {9}. Participants who are randomized to the exercise intervention will be given the necessary equipment to participate either before they depart the site or by mail. Each participant will take part in the study for 16 weeks, with a total of three onsite in-person visits: one at baseline, one interim visit at week 8, and a final visit at the end of the 16-week intervention.

The primary objective of this study is to measure the effect of combination administration (NR + exercise) on skeletal muscle quality (strength along with mitochondrial OXPHOS capacity) in AYA HCT survivors {7}. Skeletal muscle quality will be measured by dynamometry, while OXPHOS capacity will be assessed through creatine chemical exchange saturation magnetic resonance imaging (CrCEST MRI). The secondary objective of this study is to measure the effect of combination administration (NR + exercise) on aerobic capacity (VO_2_max), as determined by cardiopulmonary exercise testing (CPET).

### Participants and randomization

**Fig. 2:**
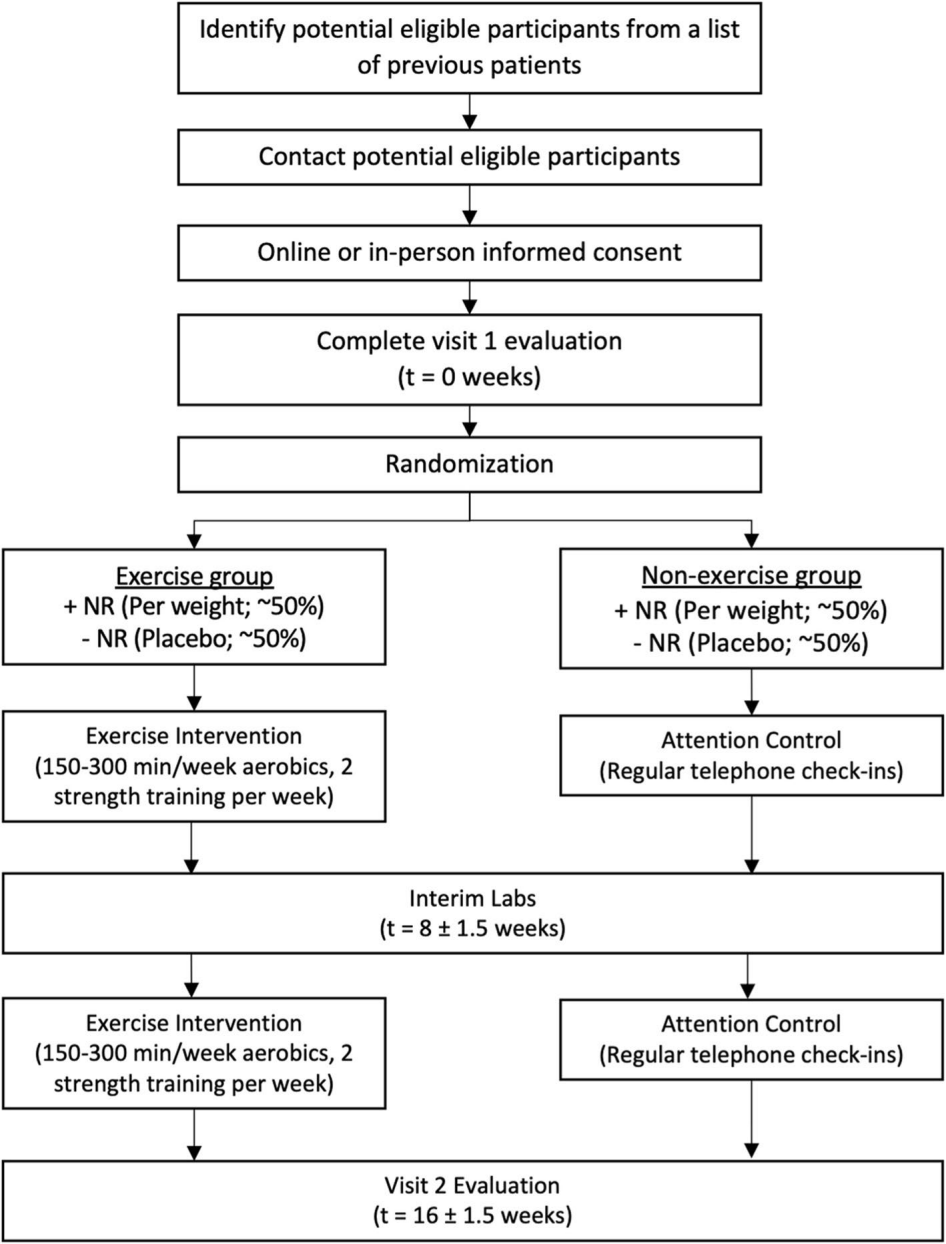
Overview of the study scheme. During their baseline visit, participants will be randomized into either exercise or non-exercise group and either NR supplementation or placebo. After randomization, participants who were randomized into exercise group will receive their equipment and be oriented to the in-home exercise intervention. Participants who were randomized into NR group will receive NR daily at a dose based on body weight: 300 mg for individuals with weight 24 to < 48 kg, 600 mg for those with weight 48 to < 72 kg, and 900 mg for those with weight 72 kg or greater. After 8 weeks ($\pm 1.5$ weeks), all the participants will have their interim visit. After 16 weeks from the baseline visit, participants will have their final study visit. The figure was generated using Microsoft PowerPoint (Version 16.62)

Potentially eligible participants will be identified and recruited both at CHOP and COH through existing databases and physician referrals. Study staff will pre-screen potential participants by reviewing the medical charts and exclude those who do not meet inclusion/exclusion criteria {15}. Individuals who meet the eligibility criteria (Table 2) will be invited to enroll. All participants will be enrolled when they provide written consent to the principal investigator(s) either virtually or in-person and all the initial eligibility requirements are met {26a}. Once eligibility is confirmed via in-person screening procedures, participants will be randomized into one of four arms, with randomization stratified by age (≥ 18y or < 18y) and site (CHOP or COH) by a designated study member using REDCAP randomization scheme (Fig. 2) {16b} {16c}. Balance between arms will be maintained through randomly permuted blocks of size four or eight {16a}. The research team, outcomes assessors, care providers, and participants will be blinded with respect to whether participants are receiving NR vs. placebo. NR and placebo are dispensed in identical capsules to avoid un-blinding. The outcomes assessor (but not the study team, care providers, or participants) will be blinded with respect to whether individuals are receiving the in-home exercise intervention vs. no exercise intervention {17a}. The Investigator will be unblinded only if an AE occurs that is determined to be serious, and treatment for the adverse event (AE) depends on knowing the group assignment. Participants/families will be unblinded at the completion of the entire study {17b}. The current study has been approved by the Institutional Review Board of Children’s Hospital of Philadelphia (approval number: IRB #17,320) and has been pre-registered at www.clinicaltrials.gov (NCT05194397). The Standard Protocol Items: Recommendations for Interventional Trials (SPIRIT) guideline was used to generate the report [27].

**Table 2:** Inclusion and Exclusion Criteria {10}

Inclusion Criteria	Exclusion Criteria
1. Males and females, ages 15–30 years at enrollment 2. Able to understand and speak English (in order to appropriately follow the intervention) 3. Diagnosis of acute leukemia (myeloid, lymphoid) requiring allogeneic HCT 4. 6–24 months from allogeneic HCT 5. Females must have a negative urine/serum pregnancy test and must use an acceptable method of contraception, including abstinence, a barrier method (diaphragm or condom), Depo-Provera, or an oral contraceptive, for the duration of the study 6. Parental/guardian permission (informed consent) and if appropriate, child assent	1. Known sensitivity to NR 2. Concurrent use of any medications, including statins, likely to increase risk of NR toxicity {11d} 3. Active malignancy, investigational agent(s) within 4 wks, or systemic glucocorticoids within 12 wks 4. Currently meeting public health exercise guidelines 5. Use of NAD^+^ precursors (supra-physiologic) within 4 weeks 6. Hemoglobin < 10 g/dL 7. Platelets < 50 K 8. Diabetes Mellitus requiring insulin or insulin secretagogue 9. HbA1C ≥ 8% 10. Kidney disease (eGFR < 60 ml/min/1.73 m2) 11. Liver disease (ALT/AST > 3 × ULN) 12. Limitations in physical function preventing exercise testing/training 13. Contraindications to MRI 14. Unstable angina or history of acute myocardial infarction (< 5 days of planned study procedures) 15. Recurrent syncope 16. Symptomatic severe aortic stenosis 17. Uncontrolled arrhythmia causing symptoms 18. Pulmonary embolus < 3 months of study procedures 19. Thrombosis of lower extremities 20. Symptomatic moderate or severe persistent asthma based on FEV from pre-HCT pulmonary function testing 21. Room air desaturation at rest ≤ 85% 22. Females: Pregnant or planning pregnancy 23. Non-cardiopulmonary disorders that may affect exercise performance or be aggravated by exercise (e.g., infection, renal failure, thyrotoxicosis, > moderate GVHD resulting in physical or functional impairment) 24. Parents/guardians or subjects who, in the opinion of the Investigator, may be non-compliant with study schedules or procedures

### Study assessments {18a}

All study participants will complete a review of medical history, have their vital signs measured, and have laboratory assessments (CBC, CMP, HbA1c, lipid profile, blood to be banked for future ancillary metabolic and/or hormonal studies) (Table 3). Activity monitoring will be reviewed for seven days via an activity monitor (ActiGraph wGT3X-BT; ActiGraph Corp., Pensacola, FL) and, if assigned, the exercise training orientation will occur. After all study procedures are completed, supplement/placebo (NR/placebo) will be provided to the participant along with instructions and administration log. Specific to the primary and secondary outcomes, all participants will undergo the following assessments at the baseline and follow-up visits:

**Table 3:**
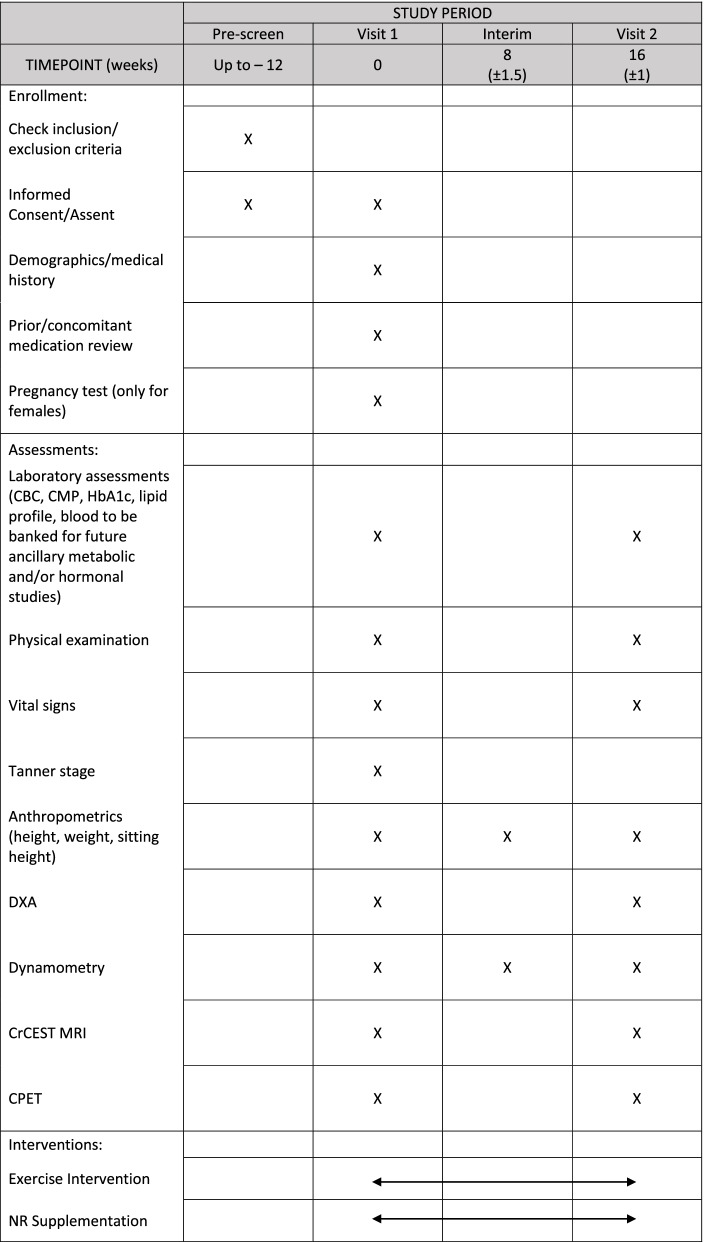
Schedule of study procedures {13}

#### Dynamometry (hand grip, lower extremities)

Trained personnel will perform testing at CHOP and COH. A dynamometer (Takei Scientific Instruments Co., Ltd., Japan) will be used to measure instantaneous hand grip strength because in epidemiological studies this index of muscle strength has been shown to be associated with morbidity and mortality [28]. Three trials will be performed using each hand, and the maximum value will be recorded (N). The Biodex Dynamometer (Biodex Corp., Shirley, NY) will be used to measure muscular strength in the ankle and knee. Participant will perform isometric strength tests, with three repetitions performed at each position. A 5 s rest is given between repetitions. At each position, peak torque (N) will be recorded. References will be used to generate age- and sex-specific Z-scores [29].

*CrCEST MRI* is a non-invasive way to test skeletal muscle OXPHOS capacity [30]. With CrCEST, creatine content can be simultaneously measured in every muscle of the leg [31, 32]. CrCEST is used to determine the exponential time constant for the decline in creatine post-exercise (τCr) in each muscle of the calf (lateral gastrocnemius, medial gastrocnemius, and soleus) after an in-magnet standardized exercise on an MR-compatible. Prolonged τCr is suggestive of decreased OXPHOS capacity. In a “proof-of-principle” study using CrCEST at a 7 T MRI field strength [33], our team found that individuals with genetic diseases affecting mitochondria had significantly prolonged post-exercise τCr in the medial gastrocnemius muscle, indicating decreased OXPHOS capacity. Using CrCEST, we also found that habitual exercise duration is positively associated with muscle OXPHOS in individuals with genetic mitochondrial disorders [33]. In a separate study using CrCEST at 3 T, we found that decreased physical activity in individuals with Friedreich’s Ataxia, a neurological disorder, accounted for some of the difference in τCr in lateral gastrocnemius between individuals with Friedreich’s Ataxia and healthy controls [34]. We will use the same 3 T CrCEST to examine the effects of mitochondria-targeted interventions in the present study.

#### *CPET*

Maximal oxygen uptake (VO_2_max) derived from CPET represents the integrative efficiency with which oxygen is delivered (by the heart and vascular system) and used (primarily by skeletal muscle) to produce ATP to sustain exercise. Using near infrared spectroscopy, an association has been found between muscle oxygen extraction and CPET performance, specifically implicating muscle mitochondrial deficits in individuals with low VO_2_max [35]. Study participants will undergo an incremental symptom-limited CPET. After a 1-min warm-up at 0-Watt (W) workload, a ramp protocol of 5 or 10 W/min will be started and continued until maximal volition. Participants will be monitored for any potentially exercise limiting symptoms such as chest pain, signs of ischemia, or arrhythmias to develop or other indications for exercise termination to appear. Respiratory gas exchange measurements will be obtained breath by breath by a commercially available system at CHOP and at COH (Vmax 29C; Sensormedics, Yorba Linda, CA). VO_2_max will be recorded as the mean value of VO_2_ during the last 20 s of the test. Gas exchange will be measured continuously throughout exercise and for the first 2 min of recovery. The ventilatory anaerobic threshold will be detected by use of the V-slope and dual criteria methods. Musculoskeletal aerobic work efficiency will be calculated as the change in VO_2_ versus the change in work rate (ml O_2_/min/Watt) prior to anaerobic threshold (AT).

### Intervention components

#### *Exercise*

Participants randomized to exercise intervention group will be provided with the results of their CPET and dynamometry and receive an individually tailored exercise prescription, written by the exercise physiologist. The participant will be given a set of weights (varying depending on their weight and physical ability), a foldable exercise bike, (FB350 Folding Exercise Bike; XTERRA Fitness, Jonesboro, AR), resistance bands, a tablet (iPad 9^th^ Generation; Apple Inc., Cupertino, CA) to interact with the exercise physiologist, and a heart rate monitor (Polar Verity Sense; Polar Electro Oy, Kempele, Finland). Those randomized to the “no exercise” intervention group will be given the option to receive a similar individually tailored exercise prescription after the 16-week intervention is complete. The exercise prescription will be based on the results of their baseline assessments and account for any functional impairments. Each prescription will include aerobic and strengthening components designed to progress persons gradually towards the 150–300 min of aerobic activity (with duration depending on intensity) and twice weekly strengthening over 16 weeks that are recommended as part of public health guidance [36].

The exercise physiologist will supervise and provide guidance for the participants six times for the first two weeks, and then taper to twice a week for weeks 3–4, once for weeks 5–7, every other week for weeks 8–11, and to one time midway between weeks 12–16, each visit expecting to last one hour. During each session, participants will be asked to answer general physical and mental health questions. For aerobic training, the eventual intensity and duration goal will be for participants to train at 70–80% of peak heart rate 20–45 min 3–5 days/week [37]. Participants who have blunted heart rate responses to exercise will train using the Borg Rating of Perceived Exertion (RPE) scale [37]. For resistance exercises the goal will be a load/weight that results in fatigue after 3 sets of 10–12 repetitions on 8–10 exercises 2 days/week [38]. As many of these individuals will have chronic conditions and/or will be sedentary at baseline, the initial intensity and duration of the training may be much lower than typical sedentary individuals. In addition, progression toward and achievement of this goal will be heterogeneous. However, our tailored approach has great potential to impact our outcomes of interest since individuals who are sedentary [39] and those who are the most impaired may be positioned to achieve the largest incremental change [40]. Tailoring will be designed to allow each participant to start at a level reflecting their initial capacity, with personalized modifications as the study continues. Progression will also be tailored and be modified during the study to accommodate response to the intervention {11b}.

#### NR Supplement dose and rationale for dose selection

We will use 300 mg tablets of the supplement nicotinamide riboside (NR) (Tru Niagen®, ChromaDex, Irvine CA) which is the formulation used in previous well-controlled pharmacokinetic/pharmacodynamic and interventional studies [19, 41]. NR is a form of vitamin B3, a precursor for NAD^+^ [14]. NR is available as a dietary supplement and is generally recognized as safe (GRAS) by the FDA.

The “no adverse effect level” of NR proposed by the European Commission Scientific Committee on Food was 25 mg/kg per day, and the conservative tolerable upper intake level proposed was 12.5 mg/kg per day, or 900 mg for adults [42]. In adults, doses of 100, 300, and 1,000 mg of NR achieve dose dependent increases in the blood NAD^+^ metabolome [41], thus we propose to use the tolerable upper intake level, 900 mg, as the maximum dose for adults, and corresponding doses (in proportion to body weight) in children. We will use the following doses:

- For individuals with weight 72 kg: 900 mg po qd × 16 wks.
- For individuals with weight 48 to < 72 kg: 600 mg po qd × 16 wks.
- For individuals with weight 24 to < 48 kg: 300 mg po qd × 16 wks.

This regimen achieves a maximum dose of 12.5 mg/kg/day (24 kg is ~ 3rd%ile for weight for a 10yo child, thus this weight threshold is more than sufficient for adolescents ages 15y and older to enroll). In a previous pediatric study, ~ 1,200 mg/m2 of nicotinamide for 5y was well-tolerated, more than what is proposed here [21] {11a}.

A concurrent use of any medication that likely increases the risk of NR toxicity is not permitted for all participants during the study period. Furthermore, those who are using such medications will be excluded from the study {11d}.

### Data management {19}

The study team will work with Data Management CHOP CHPS Informatics Core to generate case report forms and design a REDCap database for data capture. REDCap is an NIH-supported web-based data management software designed by Vanderbilt University investigators.

The PI is responsible for the accuracy and completeness of data collection and management. The PI may designate qualified individual(s) to collect data and manage data. Only investigators and research staff that have completed appropriate IRB training and approval and are listed on the IRB approved protocol are eligible to collect and work on information from the study. Future studies that may use patients or data collected from this study must have separate approved IRB protocols and consent forms, if applicable. Partial or complete datasets will be provided depending on the stated purpose of each data request. Only de-identified data will be shared. We will make the data available to users only under a data-sharing agreement that provides for:

- commitment to use the data only for not-for-profit research purposes;
- commitment to maintain the data in a secure environment;
- commitment to protect the privacy and identity of study participants and not to identify any individual participant;
- commitment to securing the data using appropriate computer technology;
- commitment to destroying or returning the data after analyses are completed;
- commitment that the data will not be transferred to other users;
- commitment to maintain all appropriate regulatory requirements;
- commitment to acknowledge the source of the data (NIH grant support and research team) in all publications and presentations.

It is the goal of this research team to translate the study findings into clinical care in order to impact the health and well-being of children. To that end, it is the intention of the research team to communicate the results of this study to the scientific community as rapidly as possible, and to share data as openly as possible {31a}.

Recruitment data will be recorded onto the screening questionnaire. Original data will be recorded directly onto CRFs by the study coordinator or a study investigator. Copies of testing results will be received through inter-office mailing, picked up directly, and sometimes through email. This information also will be recorded onto CRFs while the originals may be kept at the testing site. CRFs will be kept in a locked filing cabinet in a locked room at all times (study coordinator and/or PI office). All information will be transferred to REDCap, a secure, web-based application supported by the CHOP Research Institute. The password to log onto the database will be unique to each member of the study team. Information may also be stored in Oncore Clinical Trials Management System, a secure electronic data capture system with access controls and a data back-up plan. The system is password protected. Only study team members will have access to study data and case report forms stored in OnCore. Access to the system is monitored and logged for review, if needed {29}. The identifiable information collected as part of this study will be retained for a duration that is compliant with CHOP Data Retention Policy A-3–9 {31c}. Blood specimens will be retained indefinitely, as subjects will consent to during informed consent. The PHI linked to these specimens will remain coded and accessible only to IRB-approved study staff {27}.

Once a year, the division of Oncology at the CHOP will audit the study. Additionally, the CHOP IRB will, at random, select the study for an internal audit. The study will follow the institutional guideline to ensure that it meets the regulations and is prepared for both an internal and an external audit {23}.

### Harms management {22}

Safety will be monitored through the collection of laboratory assessments at baseline and follow-up. Participants will also be monitored using a standardized assessment of symptoms. We will use the Common Terminology Criteria for Adverse Events (CTCAE, version 5.0, U.S. Department of Health and Human Services) to grade AEs. Individuals who experience a new Grade 3 or higher AE that is at least possibly related to the study intervention may require cessation of study participation at the discretion of the investigative team. Grade 4 AEs will require study cessation of study participation.

If any unanticipated problems related to the research involving risks to subjects or others happen during the course of this study including serious adverse events (SAE), they will be reported to the IRB in accordance with CHOP IRB SOP 408: Unanticipated Problems Involving Risks to Subjects. AEs that are not serious but that are notable and could involve risks to subjects will be summarized in narrative or other format and submitted to the IRB at the time of continuing review. All AEs will be noted in the study records and on the case report form with a full description including the nature, date and time of onset, determination of non-serious versus serious, intensity (mild, moderate, severe), duration, causality, and outcome of the event. An AE is defined as any untoward medical occurrence in a subject who has received an intervention (drug, biologic, or other intervention), and an SAE is defined as any adverse drug experience occurring at any dose that results in death, in a life-threatening event, in a persistent or significant disability/incapacity, or a congenital anomaly/birth defect in the offspring of a subject. The occurrence does not necessarily have to have a causal relationship with the treatment. An AE can therefore be any unfavorable or unintended sign (including an abnormal laboratory finding, for example), symptom, or disease temporally associated with the use of a medicinal product, whether or not considered related to the medicinal product.

The Safety Monitoring Uniform Report Form (SMURF) will also be used to assess NR specific side effects and will be administered at each in-person visit. The full SMURF contains a general inquiry, several questions about daily activities (e.g., sleep, appetite, energy level, bowel and bladder function), and modified queries specific to NR side effects. The general inquiry includes an open-ended question about any problems or complaints, as well as questions regarding the need for other medications and doctor or health care encounters since the last study visit. The next section includes 25 system-specific queries to ensure completeness. All new AEs will be documented. We will also check on the status of any previously reported possible AEs.

### Statistical considerations

Linear mixed-effects modeling will be used to evaluate effect of interventions over time on the primary and key secondary outcomes. The models incorporate subject-specific random effects to account for within-subject correlation due to repeated measures. Group averages, as well as subject-specific intercepts and slopes, will be estimated to capture potential variations in the baseline values and slopes among individuals. Linear mixed-effects models accommodate missing data due to dropout, such that all randomized participants can be included. We will compare participants who complete the study with those who do not and identify potential factors that are associated with dropout {20c}. In the mixed-effects models, we will adjust for time-invariant covariates (age at randomization, sex) and time-varying covariates (muscle strength, lean mass, muscle OXPHOS capacity, VO2max). By including these indices of “muscle quality” as covariates in the models, we will be able to assess whether intervention effects are explained by these effects. To assess other potential factors influencing outcomes, we will also analyze the incremental effect of adding each of the other measured covariates (final dose of NR achieved, final training intensity achieved) to the models.

The primary outcome for our study is the change in muscle strength (quadriceps extension, Z-score) from baseline to 16 weeks. We expect exercise and NR to each produce an increase (relative to control/placebo) with respect to the primary outcome of muscle strength from baseline to week 16. We posit that the effects of exercise and NR in combination will be additive and possibly even synergistic. Despite our strategies to track and optimize adherence, it is possible that the change in Z-score will vary across participants in response to one or both interventions. We will fit a linear model with the outcome of change in Z-score of muscle strength and covariates of treatment arm and baseline Z-score. Using a modification of previous strategies for the analysis of factorial designs, the family-wise Type I error rate is maintained at < 5% by using a two-stage approach: a Holm-Bonferroni correction is applied in Stage 1, and a single contrast is pre-specified in Stage 2 [43]. In Stage 1, we will test if the mean Z-score in each of the 3 active treatment arms differs from control (no-exercise + placebo). In Stage 2, if the exercise + NR arm differs from the control arm, we will then compare exercise + NR to exercise alone, to determine whether NR increases mean Z-score beyond the effect of exercise alone; 95% confidence intervals will be determined for each effect of interest.

In secondary analyses, we will seek to identify disease covariates and physiological factors (e.g., low muscle OXPHOS capacity at baseline) that are associated with treatment response [44]. With respect to additional outcomes, we anticipate that strength in other muscle groups, lower extremity muscle mass (measured via DXA), and muscle OXPHOS capacity (measured via CrCEST), will all increase in response to both interventions, and in statistical models will demonstrate independent contributions to the observed increases in muscle strength (primary outcome) and other key outcomes. In addition, blood metabolomics will show correlates of increased OXPHOS capacity (e.g., decreased acylcarnitine related to improved fatty acid oxidation capacity, organic acid profiles consistent with increased TCA cycle anaplerosis, and increased CoA species). Even if we do not detect changes in response to either of these interventions, our mechanistic assessments will produce an integrated understanding of the factors associated with low muscle mass, strength and OXPHOS in HCT survivors. The safety data will be an ancillary benefit to guide clinicians caring for HCT survivors who may be considering taking this supplement. The primary analysis will be repeated with each secondary outcome. Additional models will explore whether change from baseline can be explained by either baseline demographic or clinical covariates (age at randomization, sex, key clinical characteristics such as anthracycline and/or steroid exposure, age at HCT, TBI, GvHD grades II-IV, interval since HCT, and hypogonadism) or features of the intervention (final dose of NR, final training intensity in Mets per week). We will explore interactions between key variables and treatment arm. With respect to sex, we will include sex by treatment interactions in the model and explore the results of individual sex-stratified analyses, though we may lack adequate power to detect sex-specific differences {12}.

In addition, we expect exercise and NR to each produce an increase (relative to control/placebo) with respect to the secondary outcome of VO_2_max. We expect that the effects of exercise and NR in combination will be additive, and possibly even synergistic. The effect of sex will also be assessed. The analysis for VO_2_max Z-score (Aim 2) will be the same as for Aim 1, since both outcomes are expressed as a Z-score. We will fit a linear model with the outcome of change in Z-score of VO_2_max and covariates of treatment arm and baseline Z-score for VO_2_max {8} {20a}.

### Sample size and power

With the proposed design, we have > 80% power to detect four scenarios of treatment-related changes in mean Z-score of 0.93–1.66 accounting for multiple testing with a family-wise Type I error rate < 5%. Notably, we have 80% power to detect a difference between exercise and exercise + NR (Scenario 1) of 0.73 Z-score. We have demonstrated that increases in muscle group specific strength of up to 100% (absolute amount) over 24 weeks are plausible, depending on the degree of initial deficits and muscle group tested [45]. In a 12-week pilot study, a mean increase of quadriceps strength of 14% was observed [46]. In a previous meta-analysis in adults, effect sizes of 0.53–0.79 were reported with supervised aerobic exercise [47, 48]. We anticipate that increases in the present study may be even larger for several reasons. First, these relatively young participants will have substantial muscle deficits, thus there is a clear opportunity to demonstrate benefit. Our preliminary data indicate muscle deficits in HCT survivors, with low Z-scores for muscle area (mean -0.9, SD 1.4). Also, we have chosen to intervene at the soonest potential opportunity post-HCT, thus we expect muscle deficits will be less established and participants will be more responsive to the interventions. Our team has considerable experience in supervising, individualizing, and adjusting the exercise regimen to achieve maximum benefit. Also, we will randomize 80 participants to achieve 64 (*n* = 16 in each of 4 arms) with complete data (i.e., conservatively allowing 20% attrition) {14}.

## Discussion

To our knowledge, our study represents the first intervention clinical trial to use combination of exercise and NR in AYA HCT survivors to address sarcopenia. Additionally, this trial will set the stage for the development of future studies that leverage the promise of a mitochondrial bioenergetic approach to the metabolic complications of cancer therapy. With mechanistic insights from this study, we can pursue trials to enhance clinically relevant metabolic function (e.g., glucose homeostasis that impacts risk for diabetes mellitus) in affected individuals after cancer therapy. The growing population of AYA cancer survivors (estimated to be 633,000 in the U.S. alone), makes the development of such interventions imperative, to optimize their long-term health in decades after completion of their cancer treatment [49].

We have considered a number of potential limitations and barriers that may arise in performing this study and have developed solutions in anticipation. It is possible that despite having an adequate number of potentially eligible participants (anticipated *N* = 185), we will not accrue the 80 HCT survivors over the course of the study. To address this concern, we will review and report accrual quarterly, and if recruitment is slower than anticipated, we will take several steps. Specifically, we will: 1) work with the primary treatment team to perform as many study procedures as possible during regularly scheduled clinic visit days; 2) minimize the time required for study measurements (total time commitment 6 h); and 3) provide compensation ($200) to each participant for each visit. If necessary, we can also expand the age range to 12-39y, with continued stratification of randomization by pediatric/adult status since the results may not vary significantly for those in peri-pubertal stages and those in late young adulthood.

With regards to ensuring adherence and compliance to the intervention, our team will be monitoring study participants in real-time via regular telephone check-ins and for those participating in exercise, via remote oversight, and thus be well-positioned to troubleshoot any barriers that may emerge during the conduct of the study. For NR/placebo, the investigative team will interview the adherence and intervene where necessary to ensure consistent administration of NR/placebo. Barriers to adherence can be reviewed at weekly check-ins and the interim visit. With regards to the exercise intervention, in-person orientation at the study site to exercise training using the in-home equipment will occur on the day of randomization, thus any functional or logistical limitations can be addressed in real time. To improve retention, we will offer participants randomized to the “no exercise” condition the opportunity to participate in in-home training after completion of the study {30}. Subjects who withdraw from the study will be recommended to update their medical history/concomitant medications, undergo physical exam, measure vital signs, test their clinical labs, get a DXA scan, get a 60 min MRI, perform a CPET, provide used/unused NR or placebo, and review adverse events as the early termination visit if both safe and feasible {18b}. In our team’s previous two studies of remotely supervised exercise interventions, we have demonstrated adherence rates exceeding 80% using similar procedures {11c}.

We will apply the insights gained from this trial to develop individualized, evidence-supported precision initiatives that will reduce chronic disease burden in high-risk cancer survivors. Our proposed trial is an important first step and will guide future interventions. We may also find that the benefits that accrue to skeletal muscle in response to skeletal muscle-focused interventions are recapitulated in other organ systems. For example, NAD^+^ precursors have impact on cardiac muscle, tissue that can also be adversely affected in cancer patients; in a preclinical model of dilated cardiomyopathy, NAD^+^ precursor attenuated the development of heart failure [50]. Additional metrics, such as circulatory power at peak exercise, can be measured to provide a non-invasive estimate of the circulatory system (i.e., heart rate, stroke volume, and systemic blood pressure) at peak exercise to explore such potential benefits.

## Trial status

The study recruitment has not commenced. The approximate date of the final participant recruitment is January 2025.

### Supplementary Information

**Additional file 1:** **Appendix II.** Informed Consent Form {32}.

## Data Availability

Study enrollment has not yet commenced; thus, there is no available data or materials.

## Appendix

### Data and Safety Monitoring Board charter and responsibilities {21a}

The Data and Safety Monitoring Board (DSMB) will act in an advisory capacity to the CHOP IRB to monitor participant safety, data quality and evaluate the progress of the study. It includes Scott Baker (Chair), Wassim Chemaitilly, Bonnie Ky, and Garry Cutter {5d}.

#### DSMB responsibilities

The DSMB responsibilities are to:

- review the research protocol, informed consent documents and plans for data safety and monitoring;
- evaluate the progress of the trial, including periodic assessments of data quality and timeliness, recruitment, accrual and retention, participant risk versus benefit, and other factors that can affect study outcome;
- consider factors external to the study when relevant information becomes available, such as scientific or therapeutic developments that may have an impact on the safety of the participants or the ethics of the trial;
- review study performance, make recommendations and assist in the resolution of problems reported by the Principal Investigator (s);
- protect the safety of the study participants;
- make recommendations to the Principal Investigators, the IRB, and, if required, to the Food and Drug Administration (FDA) concerning continuation, termination or other modifications of the trial based on the observed beneficial or adverse effects of the supplement under study; and
- ensure the confidentiality of the study data and the results of monitoring.

The DSMB will discharge itself from its duties when the last participant completes the study.

#### Membership

The DSMB will include (1) a pediatric oncologist with expertise in bone marrow transplantation who will serve as Chairperson, (2) pediatric endocrinologist, (3) a statistician, and (4) a cardiologist with expertise in cardiac late effects after cancer therapy. In the event of a “tie,” the DSMB Chair (pediatric oncologist) will make the determination.

#### Board process

At the first meeting, prior to subject enrollment, the DSMB will discuss the protocol, suggest any needed modifications, and establish guidelines to study monitoring by the Board. The DSMB Chairperson in consultation with the Principal Investigators will prepare the agenda to address the review of study materials, modifications to the study protocol and informed consent document, initiation of the trial, reporting of adverse events, statistical analysis plan including stopping rules, etc.

Meetings of the DSMB will be held initially every 6 months at the discretion of the Chairperson and/or the PIs and IRB; frequency of meetings may be modified as per the initial experience. An emergency meeting of the DSMB may be called at any time by the Chair other others should participant safety questions or other unanticipated problems arise.

Meetings are closed to the public because discussions may address confidential participant data. Meetings are attended by the Principal Investigators and members of their staff. Meetings may be convened as conference calls as well as in-person.

#### Meeting format

DSMB meetings will consist of open sessions and, if needed, closed sessions. Discussion held in all sessions is confidential. The Principal Investigators and key members of the study team attend the open sessions. Open session discussion will focus on the conduct and progress of the study, including participant accrual, protocol compliance, and problems encountered. Unblinded data are not presented in the open session.

Any closed session, if needed, will be attended by the DSMB members. The study statistician may be present, at the request of the DSMB. Any unblinded data, if necessary, are presented during the closed session.

Each meeting must include a recommendation to continue or to terminate the study and whether the DSMB has any concerns about participant safety made by a formal DSMB majority or unanimous vote. Should the DSMB decide to issue a termination recommendation, the full vote of the DSMB is required.

A recommendation to terminate the study may be made by the DSMB at any time by majority vote. The Chair should provide such a recommendation to the PIs immediately by telephone and email.

#### Meeting materials

DSMB interim report templates will be prepared by the study staff to be reviewed by the DSMB members. Format and content of the reports should be finalized and approved at the initial DSMB meeting, although changes throughout the trial may be requested by the Board.

The reports will list and summarize safety data and describe the status of the study. Open session reports generally include administrative reports by site that describe participants screened, enrolled, completed, and discontinued, as well as baseline characteristics of the study population. Other general information on study status may also be presented. Listings of adverse events and serious adverse events as well as any other information requested by the DSMB may also be in the open session report, but none of the data will be presented in an unblinded manner. In the closed session report, listings of adverse events and serious adverse events as well as any other information will be reported grouped by treatment arm, remaining blinded to details of the intervention. The DSMB may direct additions and other modifications to the reports on a one-time or continuing basis.

#### Reports from the DSMB

A formal report containing the meeting minutes and recommendations for continuation or modifications of the study will be prepared by the DSMB Chairperson. The draft report will be sent to the DSMB members for review and approval, and then sent to the Principal Investigators. It is the responsibility of the Principal Investigators to distribute the DSMB recommendation to all co-investigators and to ensure that copies are submitted to all the IRBs associated with the study.

#### Confidentiality

All materials, discussions and proceedings of the DSMB are completely confidential. Members and other participants in DSMB meetings are expected to maintain confidentiality.

## Abbreviations

AE: Adverse event
ATP: Adenosine triphosphate
AYA: Adolescents and young adults
CBC: Complete blood count
CHOP: Children’s Hospital of Philadelphia
CMP: Complete metabolic panel
CrCEST: Creatine chemical exchange saturation transfer
COH: City of Hope Medical Center
CTCAE: Common Terminology Criteria for Adverse Events
CVD: Cardiovascular disease
DXA: Dual energy x-ray absorptiometry
eGFR: Estimated glomerular filtration rate
FDA: Food and Drug Administration
GRAS: Generally recognized as safe
GvHD: Graft versus host disease
HCT: Hematopoietic cell transplant
HbA1c: Hemoglobin A1c
HUP: Hospital of the University of Pennsylvania
MRI: Magnetic resonance imaging
NAD^+^: Nicotinamide adenine dinucleotide
NIH: National Institutes of Health
NR: Nicotinamide riboside
OXPHOS: Oxidative phosphorylation
PGC-1α: PPARG coactivator 1 alpha
RPE: Borg Rate of Perceived Exertion
RR: Respiratory rate
SAE: Serious adverse event
SMURF: Safety Monitoring Uniform Report Form
ULN: Upper limit of normal
VO_2_max: Peak oxygen uptake/maximal aerobic capacity
