# Supplementary material for: Exercise training and NR supplementation to improve muscle mass and fitness in adolescent and young adult hematopoietic cell transplant survivors: a randomized controlled trial {1}
Source: BMC Cancer. 2022 Jul 19;22:795. doi: 10.1186/s12885-022-09845-1 (PMC9295440; doi:10.1186/s12885-022-09845-1)
Supplement: Supplementary file 1 — Additional file 1: Appendix II. Informed Consent Form {32}. [file 12885_2022_9845_MOESM1_ESM.docx]

**Appendix II: Informed Consent Form {32}**

**Informed Consent and HIPAA Authorization Form**

**Study Title:** Intensive tailored exercise training with NAD+ precursor supplementation to improve muscle mass and fitness in adolescent and young adult hematopoietic cell transplant survivors

Version Date: March 7^th^, 2022

| **Principal Investigator:** | Sogol Mostoufi-Moab, MD, MSCE | Telephone: 267-426-9725 |
| --- | --- | --- |

You, or your child, may be eligible to take part in a research study. This form gives you important information about the study. It describes the purpose of this research study, and the risks and possible benefits of participating.

In the sections that follow, the word “we” means the study doctor and other research staff. If you are a parent or legal guardian who is giving permission for a child, please note that the word “you” refers to your child.

**Study Overview**

You or your child are being asked to take part in this research study because you were diagnosed with acute leukemia (myeloid, lymphoid) and treated with allogeneic hematopoietic cell transplantation (HCT) within the past 6-24 months.

Research has shown that adolescents and young adults who are treated with HCT are at increased risk for skeletal muscle loss, so the purpose of this study is to investigate if a combination of exercise training and an exercise supplement can mitigate this effect. The supplement is called nicotinamide riboside. Nicotinamide riboside is not FDA approved for any uses in the United States.

If you agree to take part, your participation will last for about up to 44 weeks and will involve 3 study visits. You will need to take the study drug nicotinamide riboside or placebo for 16 weeks. A placebo is an inactive substance. As a participant in the research you will:

- Receive a study drug or a placebo; you will not know which.
- Receive exercise testing and training. Some subjects will not receive exercise training.
- Have 3 extra research clinic visits;
- Have a research MRI;
- Have a DXA scan
- Other research tests such as blood draws, and cardiopulmonary exercise testing

The main risks of this study are from the dietary supplement Nicotinamide Riboside (NR). These include: flushing, rash/itching, indigestion, diarrhea, and constipation.

Risks of exercise training include rapid or irregular heartbeat, falling, muscle strains or soreness, dizziness, and nausea.

You will not benefit directly from participating in this study.

If there is anything in this form you do not understand, please ask questions. Please take your time. You do not have to take part in this study if you do not want to. If you take part, you can leave the study at any time. If you do not choose to take part in this study, you can discuss treatment options with your doctor.

Please see below for additional details about the study.

**How many people will take part?**

Approximately 80 participants will take part in this study, including about 30 participants from CHOP.

**What is the standard treatment for this condition?**

Usual care for this condition includes general counseling regarding healthful nutrition and exercise during routine clinical visits.

**What is involved in the study?**

You will be randomly assigned to 1 of 4 groups: exercise alone, supplement alone, exercise and supplement, or placebo alone. You will be randomly assigned to one of the four groups (similar to flipping a coin). There is a 1 in 4 chance that you will be assigned to any of the groups. Participants who are not randomized to receive exercise training can choose to receive training at the conclusion of the main study.

**What are the study procedures?**

Some of the procedures in this study will be repeated multiple times. Tests that are part of your regular, routine medical care will continue to be performed. Additional tests may be performed if any of your initial test results are not normal. The study involves the following tests and procedures.

**Experimental Procedures:**

Dietary Supplement NR or Placebo: If you are assigned to receive the supplement or placebo, you will take one capsule every day for about 16 weeks. You must not crush, chew, or open the capsules.

Exercise Intervention: If you are assigned to the exercise intervention, you will receive an individually tailored exercise plan based on baseline testing. The exercise plan will increase in intensity until you reach 150-300 minutes of moderate aerobic exercise per week and strength training exercises twice a week over 16 weeks using an in-home bike trainer and resistance bands. All required exercise equipment, including the in-home bike trainer and resistance bands will be provided to you by the study.

Activity Monitoring: You will wear a fitness tracker to monitor your heart rate and physical activity.

**Routine Clinical Trial Procedures**:

Medical Record Review: We will review your medical records throughout the study to collect information about your medical history, current health, diagnosis, treatments, medications, and results of clinical tests.

Research Record Review: We will review and record research-only data obtained as part of previous CHOP studies you participated in.

Medical Interviews: A team member will take your medical history, along with a listing of any medications you are taking. Throughout the study you will be asked to report if you think that anything bad has happened as a result of the study.

Pregnancy Test: *For female participants:* If you are pregnant or nursing, you will not be allowed to participate in this study. All female participants will be asked to take a urine pregnancy test before starting this study. The results will only be shared with you. If you are found to be pregnant, then you will not be able to participate/continue participation in the study. If you are under 18 years old, we encourage you to share the results of a positive pregnancy test with your parents but we cannot make you do that.

Blood Tests: Blood samples will be collected to monitor your safety and check levels of NR in your blood. We will draw approximately 50mL (10 tsps.) at each study visit.

Fasting: You will need to not eat for at least 12 hours before coming for all three visits. You will be reminded prior to those visits. You may have water during the fasting period.

Physical Examination: A routine physical exam will be conducted at the baseline and follow up visits (i.e, listening to your heart and lungs, etc.) We will also measure weight, height, and vital signs, including measurements of blood pressure, heart rate and respiratory rate. Finally, for pediatric participants, a study doctor will perform Tanner staging, which is a physical examination method to measure sexual development. Tanner staging will not be performed on adult participants.

Questionnaires: You will complete study questionnaires related to your overall health and level of physical activity.

Dynamometry: Muscle strength in the hands, knees and ankles will be measured using dynamometer. For the hand-grip, you will squeeze the handle on the device with each hand and the strength of the “squeeze” will be measured. For the ankle, you will perfom strengths tests of the ankle, such as pointing and flexing your foot, and the strength of the movement will be recorded.

Cardiopulmonary Exercise Testing: You will be asked to undergo a limited heart-lung exercise test. A special bicycle will be used which allows you to pedal while sitting back. You will be tested while pedaling with your legs on the bike. Part of the study include pulmonary function test (PFT). PFT measures how well your lungs can move air in and how fast you can breathe out. You will wear a nose clip and be asked to blow as hard and as fast as you can into a tube three or more separate times. You will be monitored carefully during these procedures. The following tests will be performed to monitor your safety.

- Electrocardiogram (ECG): ECG is an electrical tracing of the heartbeat or heart rhythm using small soft pads on your chest.
- Pulse oximeter: A small probe will be placed on your finger. This will measure the amount of oxygen in your blood.

MRI: An MRI scan takes pictures of your muscles. MRI uses a combination of a large magnet, radiowaves, and a computer to produce pictures. Over the entire study, we will ask you to complete 2 MRI scans to measure how your skeletal muscles are using energy, using investigational MRI sequences that are not FDA approved There is no contrast and no sedation administered for the research MRI. Each MRI scanning session may last up to 2 hours. During the sessions, we will ask you to perform a brief light leg exercise once or twice. You will be asked to press down on a pedal, similar to a car accelerator or piano foot pedal repetitively over a 2-minute period.

DXA: A special X-ray of the body called a “DXA scan” will be completed to

measure body composition (i.e., the amount of muscle, fat, and bone) and

bone mineral density. During the DXA scan, you will be asked to lie flat

on your back on a table as the machine scans your body. The scan occurs

for less than 15 minutes.

Adverse Event Assessment: Our study team will contact you by telephone and see you at in-person visits throughout the study to discuss how you are feeling. This is to help make sure you stay safe during your participation.

Exercise Diary: You will be provided a paper diary to track your physical activity in case the exercise application is not accessible.

**Visit Schedule**

The table below provides a brief description of the purpose and duration of each study visit. In addition to these study visits, there are periodic phone check-ins from the study team to guide your participation and ensure safety.

| **Visit** | **Study Weeks** | **Main Procedures** | **Duration** |
| --- | --- | --- | --- |
| Screening Visit | Up to 12 weeks before Visit 1 | Review Inclusion/Exclusion Criteria | 30 minutes |
| Visit 1 | Week 0 | Informed Consent/Assent, Review Inclusion/Exclusion Criteria, Medical History, Physical Exam, Pregnancy Test, Blood Draw, Dynamometry, DXA, MRI, Cardio Pulmonary Exercise Testing, Randomization, Exercise Training (If applicable), Dispense Study Supplement, AE Assessment | 8 hours |
| Interim Visit | Week 8 ± 1 | Blood Draw, Dynamometry, Dispense Study Supplement | 1 hour |
| Visit 2 | Week 16 ± 1 | Physical Exam, Pregnancy Test, Blood Draw, Dynamometry, DXA, MRI, Cardio Pulmonary Exercise Testing, Supplement Compliance (If applicable), AE Assessment | 8 hours |

**What will be done with my data and specimens during this study?**

During the study, we will collect blood and urine samples from you. By agreeing to participate in the study, you agree to give these samples to CHOP for research purposes.

**Will I receive any results from the tests done as part of this study?**

Results that could be important for your clinical care will be shared with you. Those who are placed in the exercise intervention group will receive the results from the exercise and performance tests. Those who were not randomized to receive the exercise intervention will be provided exercise testing results and offered the option to complete the performance intervention after the completion of 16 weeks. We will not share other research results with you.

**What are the risks of this study?**

Taking part in a research study involves inconveniences and risks. If you have any questions about the possible risks listed below, you should talk to your study doctor or your primary care provider/family physician. The main risks of taking part in this study are discussed below.

**Risks associated with study supplement:**

The active supplement NR may cause side effects in some people. NR is a form of vitamin B3, and is available as a dietary supplement that is generally recognized as safe by the FDA.

The main risks of NR are listed in the table below. The described potential risks also include information about supplements that are similar to NR.

| **Common:**  (between 1 out of 10 and 1 in 100 participants affected) | • GI Upset: Nausea, Heartburn, Diarrhea, Constipation, Bloating   - • Rash, Itching |
| --- | --- |
| **Less Common:**  (between 1 in 100 and 1 in 1,000 people are affected) | • Flushing  • Changes in blood pressure  • Muscle Pain/Soreness   - • Excessive Sweating |
| **Rare:**  (between 1 in 1,000 and 1 in 10,000 participant affected) | • Liver Function Abnormalities or Toxicity   - • Blurred Vision - • Swelling or fluid in the eye - • Breakdown of muscle tissue that could lead to kidney damage |

While very rare, serious or life threatening reactions known as anaphylaxis can occur while taking the study drug. These reactions may include swelling of the throat or other body parts, blood pressure drops, difficulty breathing or swallowing, loss of consciousness, or death. A study physician will be available to treat any reactions, if one should occur during your study visit.

Some side effects, including the risk of an allergic reaction/anaphylaxis, may be life-threatening.

There may be other side effects that we do not know about yet. If you experience any other side effects, inform your physician or our study team. All clinically significant side effects will be immediately treated. **At any time** **during your participation, if you feel that you are experiencing ANY side effects** **from the study medication contact a member of the study team immediately.**

If you are prescribed any new drugs during the study, you must notify the study doctor. Also notify the study doctor of any new over-the-counter drugs, supplements, herbal products, and vitamins.

If you require a clinical blood test, inform the doctor or nurse that you are actively on a study regimen, as it may affect clinical test results.

Reproductive Risks: You and your partner will need to practice safe methods (such as abstaining from sexual intercourse or using a medically accepted form of contraception) to prevent pregnancy through the duration of the study. If you have questions about preventing pregnancy, the study doctor, Dr. Sogol Mostoufi-Moab, will be able to discuss your choices and methods. In addition, if you become pregnant during the study, you must immediately contact Dr. Mostoufi-Moab.

Risks of the Exercise Intervention: *There may be a risk of minor injury with exercise training, including but not limited to muscle strain and soreness.* You may feel tired during or after exercise. There is a risk of falling during or after the exercises are completed. You will complete exercise orientation to make sure you stay safe, which includes instructing you in the correct lifting technique, and in the correct stretching and breathing techniques.

***Risks associated with other study procedures:***

Medical Interviews: There are no physical risks, but in-person and telephone interviews may cause temporary discomfort or embarrassment. You do not have to answer any questions that cause you to feel uncomfortable. These will be performed in private.

Urine Pregnancy Test (Females): You may feel embarrassment or discomfort.

Blood Test: Arm pain, bruising, bleeding, blood clot formation, and in rare instances, an infection might occur at the site where blood is drawn. There is also the possibility of dizziness or fainting while your blood is drawn.

Fasting: There are no physical risks, but you may experience fatigue and feelings of light-headedness.

Physical Examination: There are no physical risks, but you may experience fatigue, feelings of anxiey and frustration, and discomfort. The exam is similar to those that are performed as part of clinical care.

Questionnaires: There are no physical risks but you might experience momentary embarrassment or discomfort. You do not have to answer any questions that make you too uncomfortable.

Dynamometry: The dynamometer tests may result in mild, temporary hand and forearm knee, or ankle and foot discomfort.

Activity Monitoring: Potential risks include discomfort associated with physically wearing the accelerometer device. You will collect data for for routine activities of daily living and shoud not anticipate any discomfort associated with activity that you routinely complete.

This study uses an application to gather information for the researchers to use as part of this study.

There is a potential risk of confidentiality of your data. Every effort will be made to keep your information confidential.

Cardio Pulmonary Exercise Testing:

- Cardiac arrhythmia- irregular heartbeat. You will be monitored by ECG, pulse oximetry and blood pressure monitoring. A medically trained staff member will watch as you complete the procedure. If you experience an irregular heart beat the test will be stopped.
- Redness or swelling- There is a small risk that this could develop from the ECG electrodes (pads) that will be placed on the chest. You may feel slight pressure when the probes are attached to your chest.
- Injury due to falls- There is the chance that you could fall off the maching while performing this procedure. Trained exercise technicians will be present and will assist you to ensure this doesn’t happen.
- Dizziness and nausea- Some people may experience dizziness and nausea with exercise. If this occurs, we recommend participants contact the study team so that additional evaluation can be performed if warranted. Supportive measures, including lying down, resting, and consuming adequate fluids may be recommended.
- Shortness of breath - You may experience shortness of breath or chest tightness while performing the breathing tests. You will be treated if this occurs. This treatment (a medication called albuterol) may cause an increase in the heart rate.
- DXA Scan: You will be exposed to minimal radiation during the DXA scan. The radiation dose is not necessary for your medical care and will occur only as a result of your participation in the study. At doses much higher than you will receive, radiation is known to increase the risk of developing cancer after many years. At the doses you will receive, it is very likely that you will see no effects at all.

MRI: There are no known physical risks associated with MRI scanning. However, MRI machines produce loud noises, which may cause discomfort and irritation. We will provide you with earplugs or earphones to quiet the noise. You may also feel uncomfortable lying inside the magnet due to claustrophobia or inability to lie still. If you become anxious, you can tell us and we will remove you from the machine.

The MRI magnet is always activated and attracts certain metal objects. Any metal object on or inside of your body may heat up, move, and/or improperly function within the scanning room. Metal objects in the room can fly through the air toward the machine (magnet) and hit those in the area. However, there are many safety measures to prevent or reduce these risks.

**Magnetic Fields Health Risks Statement: There is also a potential risk of MRI for subjects with medical implants or other metallic objects in their body. All subjects undergoing MRI scanning must complete a screening evaluation risk in advance of the study for the presence of medical implants or other foreign bodies that could pose an injury. Every effort will be made to insure that disclosed implants or foreign bodies do not pose a risk to subjects. In cases where there is insufficient information to evaluate the risks associated with an implant or foreign body, the MRI study will not be allowed to proceed.**

**Some of the MRI pulse sequences and equipment components are not FDA-approved but are considered to pose no more than minimal risk.**

Incidental Findings: It is possible that during the course of the study we will find new health problems. If this happens, we will discuss them with you and help to review your best options with your usual physicians.

**Are there any benefits to taking part in this study?**

There is no direct benefit to taking part in this study. The knowledge gained from this research may help doctors gain additional knowledge about this therapy for survivors of HCT in the future.

**Do you need to give your consent in order to participate?**

If you decide to participate in this study, you must sign this form. A copy will be given to you to keep as a record.

**What are your responsibilities?**

Please consider the study time commitments and responsibilities as a research subject when making your decision about participating in this study. You will need to follow the study doctor’s instructions, keep all study appointments, and take the study supplement

and follow the exercise regimen as directed.

**What happens if you decide not to take part in this study?**

Participation in this study is voluntary. You do not have to take part in order to receive care at CHOP.

If you decide not to take part or if you change your mind later there will be no penalties or loss of any benefits to which you are otherwise entitled.

**Can you stop your participation in the study early?**

You can stop being in the study at any time. You do not have to give a reason.

**Can the study doctor take you out of the study early?**

The study doctor may take you off of the study if:

- Your condition worsens.
- The study is stopped.
- The study supplement is no longer available.
- You cannot meet all the requirements of the study.
- New information suggests taking part in the study may not be in your best interests.

**What choices do you have other than this study?**

There are options for you other than this study including:

- Continuing to receive your usual clinical care
- Not participating in this study.
- You may discuss other options available to you with your doctor.

**What about privacy, authorization for use of Personal Health Information (PHI) and confidentiality?**

As part of this research, health information about you will be collected. This will include information from past and present medical records, study procedures and tests, and interviews that are part of this research. Information related to your medical care at CHOP will go in your medical record. This includes laboratory test results and imaging studies, except for tests that are performed for this research only. Medical records are available to CHOP staff. Staff will view your records only when required as part of their job. Staff are required to keep your information private. Information that could identify you will not be shared with anyone - unless you provide your written consent, or it is required or allowed by law. Laboratory test results will appear in your medical record with the exception of non-CLIA approved test results, which are research labs performed only for this study. We will do our best to keep your personal information private and confidential. However, we cannot guarantee absolute confidentiality. Your personal information may be disclosed if required by law.

The results of this study may be shown at meetings and published in journals to inform other doctors and health professionals. We will keep your identity private in any publication or presentation.

Several people and organizations may review or receive your identifiable information. They will need this information to conduct the research, to assure the quality of the data, or to analyze the data or samples. These groups include:

- Members of the research team and other authorized staff at CHOP, the Hospital of the University of Pennsylvania, City of Hope Medical Center, and St. Jude Children’s Hospital
- People from agencies and organizations that perform independent accreditation and/or oversight of research; such as the Department of Health and Human Services, Office for Human Research Protections.
- Groups monitoring the safety of this study (DSMB)
- The National Institutes of Health who is sponsoring this research
- The Food and Drug Administration (FDA)

By law, CHOP is required to protect your health information. The research staff will only allow access to your health information to the groups listed above. By signing this document, you are authorizing CHOP to use and/or release your health information for this research. Some of the organizations listed above may not be required to protect your information under Federal privacy laws. If permitted by law, they may be allowed to share it with others without your permission.

There is no set time for destroying the information that will be collected for this study.

Your permission to use and share the information and data from this study will continue until the research study ends and will not expire. Researchers continue to analyze data for many years and it is not possible to know when they will be completely done.

**Certificate of Confidentiality (CoC)**

A Certificate of Confidentiality (CoC) covers this research. A CoC helps protect your identifiable information and biological samples.

A CoC protects your private information from all legal proceedings. Unless you consent, information from this research study that identifies you will not be shared outside this research.

- No one can be forced to share your identifiable information or biological samples for a lawsuit.
- Your information can't be used as evidence even if there is a court subpoena.

If you consent, your data **or biological samples could be shared for:**

- **other scientific research**

The CoC does not prevent some disclosures.

- **The researchers can't refuse requests for information from those funding this research. The NIH may need information to assess this project.**
- **You can still share information about yourself. You can also freely discuss your involvement in this research.**
- **The researchers must disclose things required by law. This includes suspected child abuse and neglect, harm to self or others, or communicable diseases.**

**Can you change your mind about the use of personal information?**

You may change your mind and withdraw your permission to use and disclose your health information at any time. To take back your permission, it is preferred that you inform the investigator in writing.

Dr. Sogol Mostoufi-Moab

The Children’s Hospital of Philadelphia

Division of Oncology

34^th^ Street and Civic Center Blvd. Philadelphia, PA 19104

In the letter, state that you changed your mind and do not want any more of your health information collected. The personal information that has been collected already will be used if necessary for the research. No new information will be collected. If you withdraw your permission to use your personal health information, you will be withdrawn from the study.

**Additional Information**

A Data Safety and Monitoring Board, an independent group of experts, will be reviewing the data from this research throughout the study.

You will be informed if changes to the study are needed to protect your health. You will be told about any new information that could affect your willingness to stay in the study, such as new risks, benefits or alternative treatments.

**Financial Information**

While you are in this study, the cost of your usual medical care – procedures, medications and doctor visits – will continue to be billed to you or your insurance.

**Will there be any additional costs?**

Depending on the data plan that you have for your mobile device, you may have additional mobile as a result of the mobile application study procedures.

CHOP is providing financial support and material for all experimental procedures, as listed above, for this study. The cost of the study supplement will be paid by the study sponsor or CHOP. You will be allowed to keep the in-home study equipment, including the exercise bike, resistance bands, and other exercise gear, as well as the iPad.

The compensation provided at each visit is designed to cover the cost of travel, parking, and meals, as well as your time and efforts. If you are under 18, your compensation will be split equally between you and your parents.

Participants/Families will be compensated as outlined below:

| Study Visit | Participant/Family Dollar Amount |
| --- | --- |
| Visit 1 | $200.00 |
| Interim Lab Visit | $50.00 |
| Visit 2 | $200.00 |
| TOTAL: | $450.00 |

**Who is funding this research study?**

The National Institutes of Health is providing funding for this study.

**What if you have questions about the study?**

If you have questions about this study or how your samples/data are going to be used, call the study doctor, Dr. Mostoufi-Moab at (267) 426-9725. You may also talk to your own doctor if you have questions or concerns.

The Institutional Review Board (IRB) at The Children’s Hospital of Philadelphia has reviewed and approved this study. The IRB looks at research studies like these and makes sure research subjects’ rights and welfare are protected. If you have questions about your rights or if you have a complaint, you can call the IRB Office at 215-590-2830.

A description of this clinical trial will be available on http://www.ClinicalTrials.gov, as required by U.S. Law. This Web site will not include information that can identify you. At most, the Web site will include a summary of the results. You can search this Web site at any time.

**What happens if you are injured during the study?**

If you are hurt or get sick from something that was done as part of this study, doctors at the clinic or hospital can arrange for emergency medical care. The Hospital does not offer financial compensation or payment for injuries due to participation in this research.

You and your insurance company will be billed for the costs of any care or injuries.

If you think you have been injured from taking part in this study, call Dr. Mostoufi-Moab (267) 426-9725. She can go over things with you, let you know of resources that may be available and give you information on what you need to do.

In case of injury resulting from this study, you will not lose any legal rights by signing this form.

**Sharing Data with the National Institutes of Health (NIH)**

**Why will my data be shared with the National Institutes of Health (NIH)?**

The NIH is funding this study. The NIH’s goal is to maximize the benefits that come from the research.

The NIH repository stores genetic information and phenotypic data from many studies. The NIH then shares that information with researchers. We will send the information about you and the other participants to a repository at the NIH. The information will be de-identified (no names or other direct information about you will be included). The NIH will not be able to re-identify you or any other individual.

The NIH intends to share the collected information with other researchers for future research. The researchers who receive data must promise to keep the data confidential and to use it only for the purpose approved by NIH. They must also promise to not try to re-identify anyone.

The goal of genetic studies is to look for genetic connections that may explain how to identify, prevent, and treat health problems. For example, genetic data may be used to find out:

- Who is more likely to develop a certain illness, such as asthma, cancer, or diabetes, or a condition like high blood pressure or obesity;
- What genes affect the progress of a certain disease or condition; and
- What genes may affect treatments which now may or may not work in certain people.

**Risks Associated with Sharing Data with the NIH**

There are risks associated with sharing your data with the NIH but they are very unlikely to occur. There is only a very small chance that someone could find out that the data came from you. If that happened, it’s possible that someone could deny you a job or health insurance. Or you could experience stress, anxiety or embarrassment.

**Benefits Associated with Sharing Data with the NIH**

Sharing your information for future research will not directly benefit you. It is hoped that it will lead to a greater understanding of the interaction between genes and health. This knowledge could help others in the future.

**Controlled or Unrestricted Access**

The data about you will either be made available by the NIH through controlled access or unrestricted. Controlled access means the data are made available for other research only after investigators have obtained approval from NIH to use the requested data for a particular project. Data for unrestricted access are publicly available to anyone (e.g., The 1000 Genomes Project).

**What will be done with my data and specimens when this study is over?**

As part of the study, we will collect blood samples. We may wish to use and share this information or samples in a future study about adolescent and young adult survivors of cancer treated with hematopoietic cell transfer.

Research could occur at CHOP, or at outside institutions, which could include for profit companies. The information and samples will be given a unique code, and only the study team of Dr. Mostoufi-Moab will retain the master list that will link any of the information or samples to information that can identify you. Information that can identify you or the blood samples may be kept permanently in a repository or computer database at CHOP.

We may not ask for your consent before using or sharing your identifiable specimens or data. You will not receive any results or financial benefit from the future research done on your specimens or data. We may share your identifiable specimens or data with outside researchers who will use them for future research.

If you leave the study, you can ask to have the data collected about you removed or the samples destroyed. You can also ask us to remove information that identifies you from the data or samples. This may not be possible if your samples and data have already been shared.

**Consent to Take Part in this Research Study and Authorization to Use and Disclose Health Information for the Research**

The research study and consent form have been explained to you by:

| Name of Person Obtaining Consent |  | Signature of Person Obtaining Consent |
| --- | --- | --- |
|  |  | Date |

By signing this form, you are indicating that you have had your questions answered, you agree to take part in this research study and if you are giving permission for a child to participate in this research study, you are legally authorized to consent to the child’s participation. You are also agreeing to let CHOP use and share the health information that will be collected for this study, as explained above. If you don’t agree to the collection, use, and sharing of health information, you (adult subjects) or your child (if you are a parent) cannot participate in this study.

**NOTE:***A foster parent is not legally authorized to consent for a foster child’s participation.*

| Name of Subject |  |  |
| --- | --- | --- |
| Signature of Subject (18 years or older) |  |  |
| **If children are research subjects, both parents must sign this consent form** | | |
| Name of Authorized Representative 1 |  | Relationship to subject:  Parent  Legal Guardian |
| Signature of Authorized Representative #1 |  | Date |
| Name of Authorized Representative #2 |  | Relationship to subject:  Parent  Legal Guardian |
| Signature of Authorized Representative #2 |  | Date |
| If the second representative is unavailable, per §46.408(b) / §50.55(e)(2), explain the reason. | | |
